# Supplementary figures and images for: Arbuscular mycorrhizal fungi increase crop yields by improving biomass under rainfed condition: a meta-analysis
Source: PeerJ. 2022 Feb 1;10:e12861. doi: 10.7717/peerj.12861 (PMC8815364; doi:10.7717/peerj.12861)

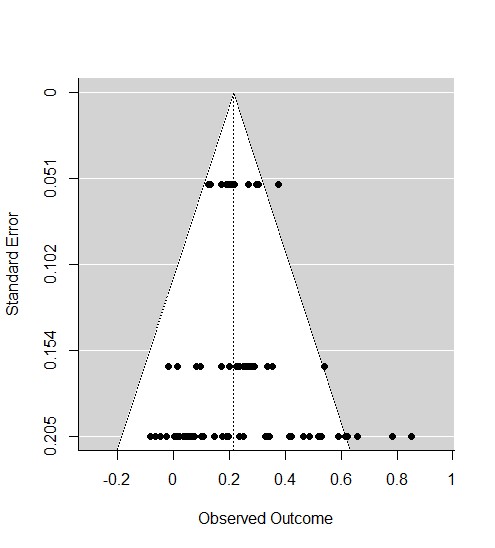

Supplement: Supplemental Information 1 [file peerj-10-12861-s001.jpg]
