# Supplementary material for: Arbuscular mycorrhizal fungi increase crop yields by improving biomass under rainfed condition: a meta-analysis
Source: PeerJ. 2022 Feb 1;10:e12861. doi: 10.7717/peerj.12861 (PMC8815364; doi:10.7717/peerj.12861)
Supplement: Supplemental Information 2 [file peerj-10-12861-s002.docx]

**Search Term Building Strategy**

**1 decide on a review question.**

**(1) formulating the initial question**

Rainfed agriculture plays key role in ensuring food security and maintain ecological balance. Especially in developing areas, most grain food are produced rainfed agricultural ecosystem. However, a major concern about rainfed agriculture is the low level of productivity, which was mainly lead by water limitation and nutrition status (Molden et al., 2011). Arbuscular mycorrhizal fungal inoculation has been concentrated widely due to its functions in improving the water status of host plant in arid ecosystem (Bryla & Duniway, 1997; Askari et al., 2019). As a natural bio-fertilizer, arbuscular mycorrhizal fungi (AMF) are paid special attraction owing to their important roles in improving nutrition of host plants and status of soil fertility (Karaca et al., 2013). So whether crop inoculation with AMF can increase yields is the question we formulating.

**(2) Conducting a scoping search**

We use “mycorrhizal”, “yield” as key words search in Google Research to determine whether there is sufficient research and indentify existing reviews, found there are two quantitative synthesis has so far targeted the AMF effects on wheat (Pellegrino, 2015) and cereal crops(Zhang et al., 2019) in field studies. However, for rainfed agriculture, AMF effects on crop yields are unavailable.

**(3) Map out the literature**

We took the studies that we found to map out the literature by the titles, abstracts and key words using the R package wordcloud2 (Figure 2).


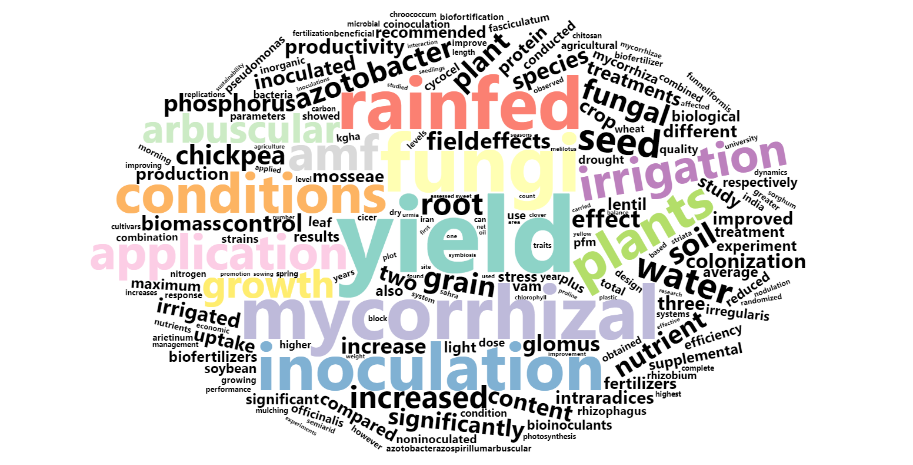


Figure 2 The word cloud of titles, abstracts and key words.

**(4) Identify question**

We assured that there was sufficient primary research for a meta-analysis based on the reviews by Pellegrino (2015) and Zhang et al. (2019) and the word cloud we made. So we decide to a meta-analysis on whether AMF can increase crop yields under rainfed condition. Here is our final PICO framework:

Population: All crops

Intervention: AMF inoculation in rainfed agriculture

Comparison/ Control group: AMF inoculation/ Non-AMF inoculation

Outcome: any yield related traits

**2. Executing the search**

We choose Web of Science as our main sources because it include multiple database such as Web of Science Core Collection, MEDLINE, SciELO Citation Index, KCl-Korean Journal Database and Russian Science Citation Index. We chose the words “rainfed” and “non-irrigat*” relevant to rainfed agriculture according to the word cloud. Similarly, we chose the words “arbuscular mycorrhizal fungi” relevant to AMF inoculation. Then we generated our initial search string as Figure 2. Then we backward and forward research from the references and the citations of literatures. Finally, we removed the duplicates and collected 72 publications for screening.


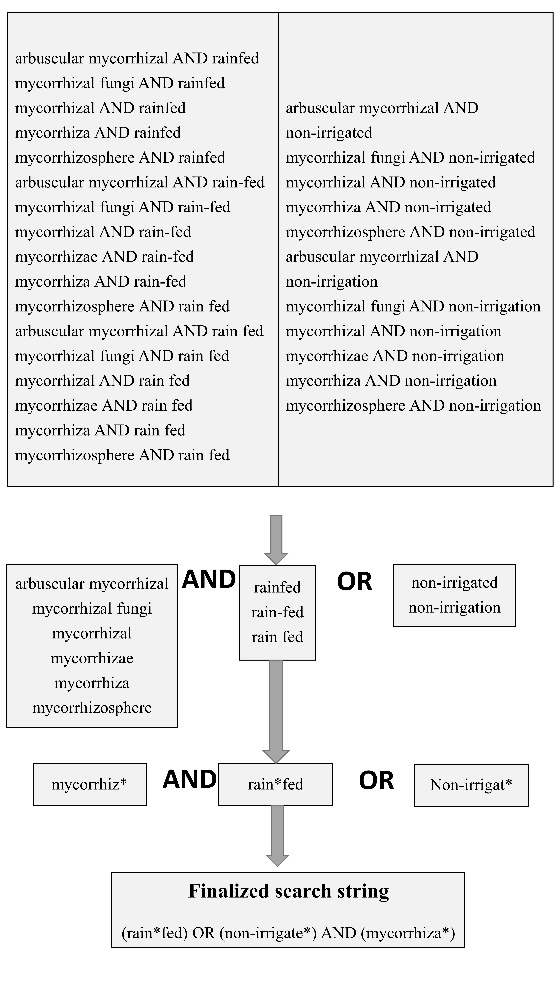


Figure 2 The process of generating the initial search string

**3. Initial screening and full-text screening**

First, we screened the literature based on their titles, abstracts and key words which excluded 21 literatures. Secondly, to ensure representativeness and accuracy of the results, the following criteria were used to screen article for inclusion: (1) the articles had to be original research; (2) field studies under rainfed conditions (3) involve an AMF treatment and a corresponding control and (4) contained replicated controlled trials. Finally, 21 articles included in the whole dataset. The Preferred Reporting Items for Systematic Reviews and Meta-analysis (PRISMA) flow diagram as flows:


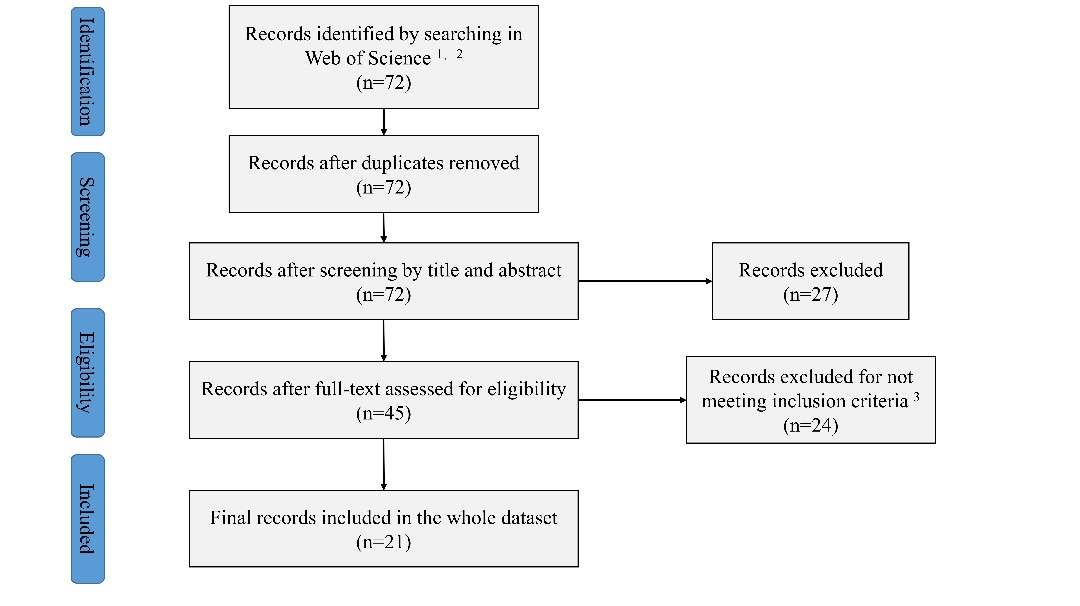


Figure 3. Preferred Reporting Items for Systematic Reviews and Meta-analysis (PRISMA) flow diagram. 1We used the terms rain*fed or non-irrigat* and mycorrhiz*, 2 the database of Web of Science Core Collection, MEDLINE, SciELO Citation Index, KCl-Korean Journal Database and Russian Science Citation Index included in Web of Science^TM^, 3 We used the following inclusion criteria: (1) the articles had to be original research; (2) field studies under rainfed conditions (3) involve an AMF treatment and a corresponding control and (4) contained replicated controlled trials.

**Reference:**

**Askari A, Ardakani MR, Paknejad F, Hosseini Y. 2019.** Effects of mycorrhizal symbiosis and seed priming on yield and water use efficiency of sesame under drought stress condition. *Scientia Horticulturae* **257**. DOI: 10.1016/j.scienta.2019.108749.

**Bryla DR, Duniway JM. 1997.** Growth, phosphorus uptake, and water relations of safflower and wheat infected with an arbuscular mycorrhizal fungus. *New Phytologist* **136**:581–590. DOI: 10.1046/j.1469-8137.1997.00780.x.

**Karaca H, Uygur V, Özkan A, Kaya Z. 2013.** Effects of Mycorrhizae and Fertilization on Soybean Yield and Nutrient Uptake. *Communications in Soil Science and Plant Analysis* **44**:2459–2471. DOI: 10.1080/00103624.2013.809730.

**Molden D, Vithanage M, de Fraiture C, Faures JM, Gordon L, Molle F, Peden D. 2011.** Water Availability and Its Use in Agriculture. In: *Treatise on Water Science*. Elsevier, 707–732. DOI: 10.1016/B978-0-444-53199-5.00108-1.

**Pellegrino E, Öpik M, Bonari E, Ercoli L. 2015.** Responses of wheat to arbuscular mycorrhizal fungi: A meta-analysis of field studies from 1975 to 2013. *Soil Biology and Biochemistry* **84**:210–217. DOI: 10.1016/j.soilbio.2015.02.020.

**Zhang S, Lehmann A, Zheng W, You Z, Rillig MC. 2019a.** Arbuscular mycorrhizal fungi increase grain yields: a meta-analysis. *New Phytologist* **222**:543–555. DOI: 10.1111/nph.15570.
