# Supplementary material for: Arbuscular mycorrhizal fungi increase crop yields by improving biomass under rainfed condition: a meta-analysis
Source: PeerJ. 2022 Feb 1;10:e12861. doi: 10.7717/peerj.12861 (PMC8815364; doi:10.7717/peerj.12861)
Supplement: Supplemental Information 5 [file peerj-10-12861-s005.docx]

**Statement of Rationale and Contribution of the work**

**1. The rationale for conducting the meta-analysis**

Rainfed agriculture occupies 80% of the world’s cultivated area, which supply 60% of crop production. Especially in developing countries, rainfed agriculture remains as the primary source of food. The function that AMF can increase the crop yield under rainfed agriculture has been testified in some crops including wheat, barley, soybean, and chickpea. However, the estimation of AMF function in rainfed agriculture is scarce on a global scale. Meanwhile, in our knowledge, the effect size of AMF was not also quantitatively evaluated in rainfed condition as in a normal grain producing system.

**2. The contribution that it makes to knowledge in light of previously published related reports, including other meta-analyses and systematic reviews.**

We explored firstly the AMF effect on crop yields under rainfed agroecosystem. Our main justification for submitting this work as an article is:

(1) AMF inoculation obviously increased crop yields by 23.0% under rainfed condition.

(2) The increase of crop yields by AMF inoculation depended on different rainfed crop groups.

(3) The yields of rainfed crops were enhanced by AMF was positive related with shoot biomass that was increased by AMF because of improving plant nutrients, photosynthesis and stress resistance.
